# Urban mosquito management administration: Mosquito (Diptera: Culicidae) habitat surveillance and questionnaire survey in Wuhan, Central China

**DOI:** 10.1371/journal.pone.0232286

**Published:** 2020-05-05

**Authors:** Xiaomin Chen, Taiping Wu, Jiansheng Liang, Liangcai Zhou

**Affiliations:** Disinfection and Vector Control Section, Wuhan Centers for Disease Prevention & Control, Wuhan, Hubei Province, People's Republic of China; Faculty of Science, Ain Shams University (ASU), EGYPT

## Abstract

**Background:**

Creating National Sanitary City (CNSC) promotes appearance, environment sanitation and public health including vector management of cities in China. However, vector management especially mosquito management and the related administrative productivity of Patriotic Health Campaign System (PHCS) of National Sanitary Cities (NSCs) were questioned by many pest control professionals and citizens. In this study, we studied mosquito management of NSCs taking Wuhan as an example. The study aimed to (1) determine the distribution and abundance of immature mosquito habitats in built-up areas of Wuhan and (2) better understand the related administration procedure in CNSC.

**Methods:**

Immature mosquito habitat surveillance was carried out in randomly selected premises of driving schools (DSs), schools or kindergartens (SKs), property management residential areas (PMRAs), construction sites (CSs), wide roads with storm drains (WRSDs) and urban creeks (UCs) in built-up areas of Wuhan from July to October 2015 followed by questionnaire interviews with one each of premise occupants and district departments responsible for mosquito management in these premises.

**Results:**

Total of 64.1 km of route were inspected in 36 DSs, 36 SKs, 36 PMRAs, 36 CSs and 36 segments of WRSD and 2,158 potential mosquito habitats with 749 (35%) mosquito-positive habitats were found. The route index (RI) was 11.7, which was 14.6 times higher than the grade C criteria for vector density control (RI = 0.8 positive habitats/km) in CNSC. Occupants of 36 DSs, 36 SKs, 36 PMRAs, 34 CSs were interviewed and 77% of them reported no difference in mosquito infestation in their premises since 2013 and 80% of them knew about the responsibility and arrangements of PHCS of mosquito management in their premises. Only 15% had the awareness of larval source reduction strategy and 14% had implemented it. Receipt the electronic/paper edition of CNSC vector management specifications from the PHCS was very low (13%) and an official notification or bulletin for rectification mosquito-positive habitats was also very low (5%). Of the 75 responsible district departments interviewed, about half (55%) reported that they had held training courses/meetings related to CNSC vector management, the majority (96%) reported that they had not carried out independent on-site supervision of premises under their jurisdiction. No differences in larval indices were found between premises which were administrative intervened and with no administrative intervention.

**Conclusions:**

The administrative intervention of PHCS had not evidently improved mosquito management of the premises in built-up areas in Wuhan. It is a violation of the original intention of the National Patriotic Health Campaign Committee in organizing CNSC programs. To combat mosquito borne diseases, and to improve the quality of life of residents, we recommend that PHCS honestly reveals defects in urban mosquito management and seriously takes those exposed defects. The PHCS should strengthen Patriotic Health Campaign activities by strict adherence to NSC standards. Further research on sustained promotion of urban mosquito management of NSCs, which focus on effective administration, as well as on improvement of related sectors of NSC standards should be carried out.

## Introduction

Vector-borne diseases continue to be a threat to people of the world [1,2]. To reduce the risk of vector-borne diseases and to improve environment sanitation, Chinese government has organized the Patriotic Health Campaign (PHC) of China since 1952. Under the direct guidance and administration of the central government, there are district and sub-district PHC in urban areas and township PHC in rural areas. The PHC Committees constitute of the hierarchical representation of vector management administrative departments of the government. The executive agencies are PHC Committee Offices (PHCCOs) and the superior of the PHCCOs are responsible for supervising, guiding and assessing vector management work of the subordinate government departments and PHC Committee Members according to government rules and regulations. District level of PHC Committee Members are responsible mainly for the administration of vector management of the industries/premises under their jurisdiction (e.g. district Education Bureaus and Housing Management Bureaus of Wuhan), while sub-district offices are responsible for other industries/premises (e.g. government authorities, enterprises and driving schools). The PHC has undoubtedly made great achievements in vector-borne disease control and public health in the history of China [3]. One of the successful programs of the PHC was eliminating the Four Evils (EFE), mainly including rodent, mosquito, fly and cockroach management which has recently evolved to be the program of vector management. A typical case of Wuhan PHC was that in the late 1990s, the deputy mayor of Shunling Gao took command of the rodent control mass campaign, which directly reduced substantially the number of cases of rodent borne hemorrhagic fever [4] of the city.

For further improvement of appearance, environment sanitation and public health of Chinese cities, the National PHC Committee has organized a program named, Creating National Sanitary City (CNSC) to carry out activities in the whole country since 1990s. Under this program a National Sanitary City (NSC) needs to meet the corresponding stringent standards in the field of organization and management of PHC, city appearance and sanitation, vector management, etc. and should establish sustainable mechanism until after CNSC (Consolidating NSC) according to the NSC standards. Mosquito management of NSCs should at least meet the grade C criteria of vector density control (Mosquito: GB/T 27771–2011). To receive the required criteria, the candidate cities have to go through several assessments including an unannounced visit and a technical assessment carried out by a group of experts every 3 years. In the unannounced visit, experts check secretly and simultaneously the status quo of the candidate cities in all fields required to be satisfied to achieve the award of NSC, the highest honor in appearance and environment sanitation and public health of China. Work by Qi et al. [5] confirmed that NSCs had significant advantage in vector management system, funds, professional staff and control effects over non-NSCs. Several authors reported that the program of CNSC had increased vector management effects in those cities [6,7]. However, vector management of CNSC is being criticized among folks of those cities as an expensive program with few benefits. They complain especially about the mosquito nuisance mainly in South and Central China. Vector management experts generally believe that the CNSC’s retrogression in vector management is quite common. Some senior vector management professionals suppose that NSCs meeting the criteria for vector density control does not truly exist, and the assessment experts are not strict in implementing the standards.

Wuhan, mega city in central China, provided us with an example to evaluate the mosquito control administration in CNSC program. Wuhan started CNSC in 1990, and failed in passing the assessments repeatedly, although substantive progress had been made in all NSC fields. EFE services, mainly rodent and mosquito control in urban old residential areas without property management were funded by the government and Wuhan Pest Control Association was authorized by Wuhan PHCCO to supervise the service quality each year from 2003 to present. There was no substantive sustainable mosquito management mechanism in other types of premises until intensive program of CNSC was initiated in March 2013. Wuhan Centers for Disease Prevention and Control prepared CNSC vector management specifications (CNSCVMS), a concise set of instructions for various type of premises which stressed the importance of larval source reduction in mosquito management [8–10]. Each urban premise was required to implement mosquito management according to CNSCVMS since 2013. The Wuhan PHCCO distributed CNSCVMS and Wuhan urban pest management regulations (WUPMR) to the district PHCCOs, and the responsible departments were required to distribute these documents to premises under their jurisdictions. Mosquito management programs in premises in each district were assessed by quarterly unannounced assessments and monthly visual assessments (a technical assessment) by third-party experts [11]. The final technical assessment was done by the experts of National PHCCO in September 2014. Going through all these assessments, the City of Wuhan was awarded NSC title in March 2015.

This study aims to evaluate the mosquito management in the built-up areas of Wuhan city with specific objectives to determine the distribution and abundance of immature mosquito habitats [12] and to better understand mosquito management administration in Wuhan program of CNSC to make a sustainable mosquito management mechanism.

## Materials and methods

### Study areas

**Fig 1:**
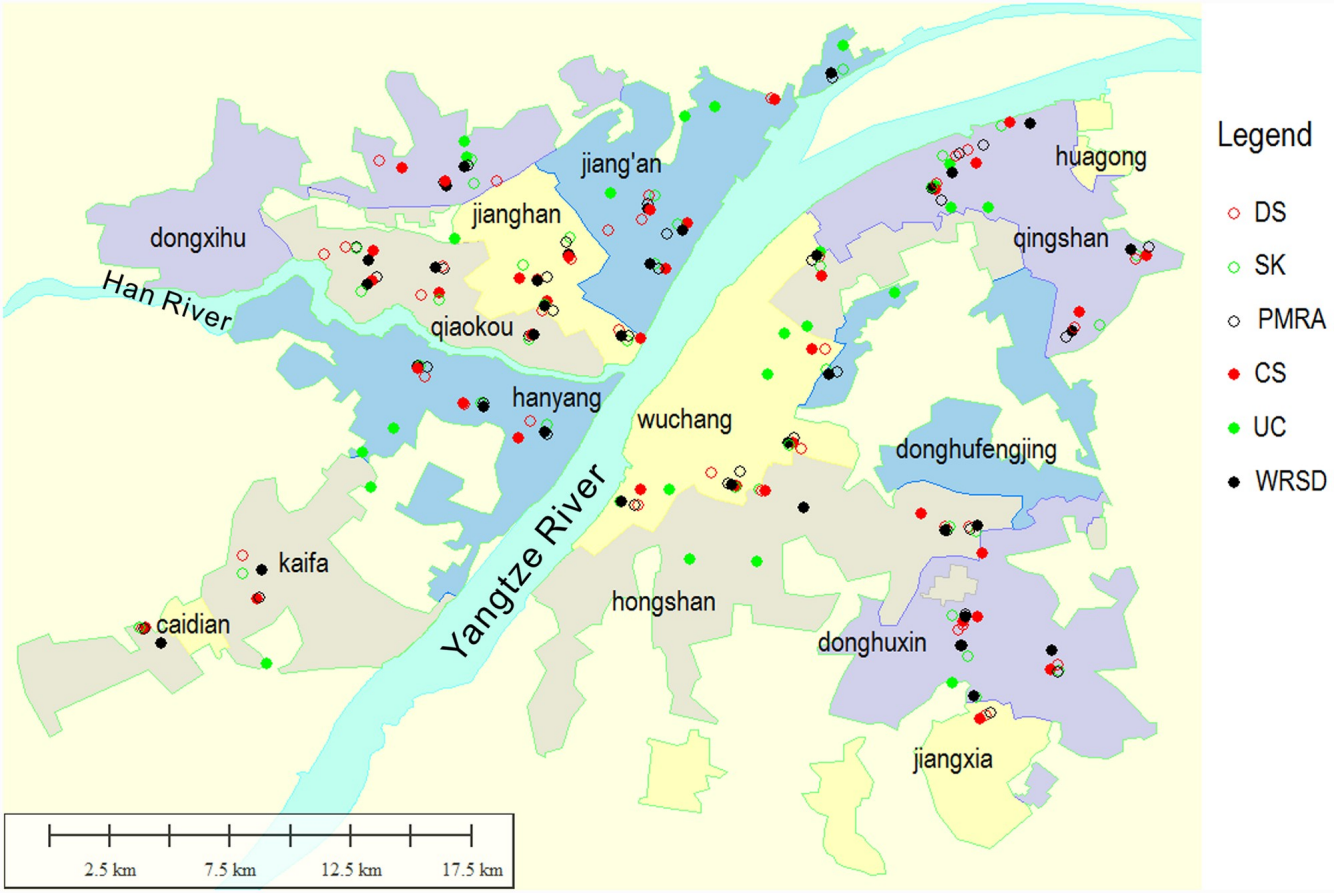
Locations of the study sites for larval surveys in built-up areas of Wuhan.

Built-up areas of Wuhan (S1 Fig) cover a total land area of 1,467 km^2^ and lie between latitude 30°23′ and 30°42′ N and longitude 114°04′ and 114°30′ E. The climate is humid subtropical with the 15.8°C to 17.5°C annual mean temperature and 1,150 to 1,450 mm annual precipitation which are suitable for mosquito breeding. Mosquito activity season is usually from May to October. Common mosquito species in urban Wuhan are *Culex quinquefasciatus* Say and *Aedes albopictus* (Skuse) [13–15]. The built-up areas consist of 14 administrative districts and stretch across the Yangtze River. Four central districts (Jiang′an, Jianghan, Qiaokou, and Hanyang), a functional district (Kaifa) and 2 new districts (Dongxihu and Caidian) lie to the Northwest of the River. Three central districts (Wuchang, Qingshan, and Hongshan), 3 functional districts (Donghuxin, Donghufengjing, and Huagong) and a new district (Jiangxia) lie to the Southeast of Yangtze River (Fig 1, S2 Fig). Although the scope of CNSC program includes communities of the Sub-districts where the new district (e.g. Xinzhou, Huangpi) government are located (S1 Fig), these areas were not selected for this study. Central districts are considered more important than the other districts by the local authority.

Driving schools (DSs), schools or kindergartens (SKs), property management residential areas (PMRAs), construction sites (CSs) [16], and wide roads with storm drains (WRSDs) [17], are administered by Sub-district Offices, district Education Bureaus, Housing Management Bureaus, Construction Management Stations, and Water Authorities, respectively. These premises are usually common with mosquito breeding habitats. Urban creeks (UCs, administered by district Water Authorities) although less common, often produce a huge number of immature mosquitoes [18].

### Sampling of premises

A sampling grid of 36 squares with a side length of 400 m was centered on random points [19,20] calculated using random longitudes and latitudes of a KML file [21] of Wuhan built-up areas border, provided by Wuhan Land Resources and Planning Bureau. Then the file of sampled squares was imported to OruxMaps in android mobile devices [22]. In each square, a DS, a SK, a PMRA, a CS and a segment of WRSD, each with 5 stagnant waters, were selected for mosquito habitats. In principle, premises closer to the center of the square were given priority to be sampled. A total of 36 DSs, 36 SKs, 36 PMRAs, 36 CSs, 36 segments of WRSD and 24 segments of UC were sampled (Fig 1, S2 Fig). Of them 9 DSs, 9 SKs, 9 PMRAs, 8 CSs, and 8 segments of WRSD were in non-central districts. When the number of UCs in built-up areas of a district was greater than the number of random points, only a number of UCs equal to the number of random points were sampled. In other cases, all UCs in built-up areas of the district were sampled. One segment, which was easy to reach and nearer to the square, of each sampled UC was selected for inspection.

### Surveys

#### Entomological survey

Immature mosquito surveys were conducted from July to October 2015 by 2 to 5 experienced vector control technicians. Potential breeding habitats were categorized as small containers (less than 37 cm in diameter), cisterns (generally made of concrete), car tires, storm drains, ditches, shallow waters (2–8 cm depth), artificial objects (such as leather shoes, toys, etc.), large containers (equal to or larger than 37 cm in diameter), pits or puddles, manholes or cable trenches, stumps (bamboo stumps or the rooted remains of metal tubes), potted (or aquatic) plants, large and medium waters (LMW—water body with an area larger than 200 m^2^, including UCs) and septic tanks.

Searches for stagnant waters were conducted mainly outdoors and tracked in the log of Oruxmaps with GPS. Route length and elapsed time of inspection in each premise were recorded in Oruxmaps as well as in paper sheets. Underground parking lots [23], sanitary corners and ends of the research premises, where positive mosquito habitats were more likely to be found, were given priority during inspection. When inspecting a premises with a small area or with less than 5 stagnant water collections, the entire outdoor area was searched. SDs, LMWs and UCs were inspected with a 500 ml dipper [24,25]. Five dip samples (1 dip per 10m) were taken from each segment of UC. All larvae and pupae in the dipper were counted. According to the national guidelines, waters of less than 200 m^2^ (e.g. small containers, tires) were just checked for the presence or absence or mosquito larvae and/or pupae but counts were not taken.

Three larval indices, container index (CI, defined as the percentage of mosquito habitats that are positive for immature mosquitoes) [26], route index (RI, defined as the number of mosquito-positive habitats per km of inspection route) and time index (TI, defined as the number of mosquito-positive habitats per hour of inspection) [22] were calculated for each premise type. LMWs were excluded in the above calculation. For LMWs, dip index (DI, percentage of mosquito-positive dips) and the average number of immature mosquitoes per positive dip were used in this study according to the criteria for vector density control of NSC.

#### Questionnaire survey

Questionnaire-based surveys were carried out in DSs, SKs, PMRAs and CSs together with entomological surveys. Face-to-face interviews were carried out with the heads of premises, health leaders or responsible persons of pest control or any other occupant of the premises who knew about their program of EFE. The interviewers used a pre-tested questionnaire to obtain information on interviewees’ knowledge about mosquitoes [27], mosquito infestation in their premises and management efforts, guidance and supervision of PHC System (PHCS) on mosquito management. Inquiries were made on mosquito management supplies (including insecticides recommended and banned by the PHCCO) and the interviewers checked those supplies when the respondents were supportive. If the premises had contracted with professional pest control companies for mosquito control, those companies were telephone interviewed later over related matters. Occupants of all DSs, SKs, PMRAs and CSs sampled except 2 CSs responded for the questionnaire while the occupants of most of WRSDs and UCs were not because they could not be found near the premises and their responsible departments were generally reluctant for such surveys.

These questionnaire surveys were followed by phone [28] and QQ (Ten cent instant messaging software) interviews with all responsible district departments, including 31 Sub-district Offices, 11 district Education Bureaus, 12 district Housing Management Bureaus, 10 district Construction Management Stations, and 11 district Water Authorities. An electronic version of an official letter with Wuhan PHCCO seal explaining the purpose of the investigation and the confidentiality of the information was sent through QQ to the responsible departments before the interviews to acquire the reliability of information. The interviews were made with heads of departments, health leaders, vector management liaison officers or any other occupants who knew about their programs of NSC vector management. The questionnaire interview included endorsement of responsibility for supervision on related premises sampled and vector management supervisions on their premises from 2013 to 2015.

### Ethical considerations

The study protocol was reviewed and approved by Wuhan PHCCO. Consent was given verbally from administrative officials of district PHCCOs, the head of the premises and the responsible departments, as this was consistent with local practice and customs for entomological and questionnaire survey, and consent was recorded by the survey staff at the time of the survey.

### Data analysis

Data were analyzed using SPSS 16.0 for Windows. The proportion of habitats were compared between premise types, the CIs were compared between habitat types using chi-square tests. Some habitat types were truncated to facilitate statistical analysis. Mean RIs, CIs and TIs were compared using non-parametric Mann-Whitney tests. Standard errors are reported.

## Results

### Mosquito habitats and larval indices

Mosquito-positive habitats were found in 84% of the sampled premises except UC. The total number of potential mosquito habitats in the 5 types of premises was 2,158. Immature mosquitoes were found in 749 habitats and the total CI was 35%. The total inspection route length and elapsed time in the study area was 64.1 km and 70.2 hrs, respectively. Total RI was 11.7 which was 14.6 times higher than the grade C criteria (RI = 0.8 positive habitats/km) and total TI was 10.7.

**Fig 2:**
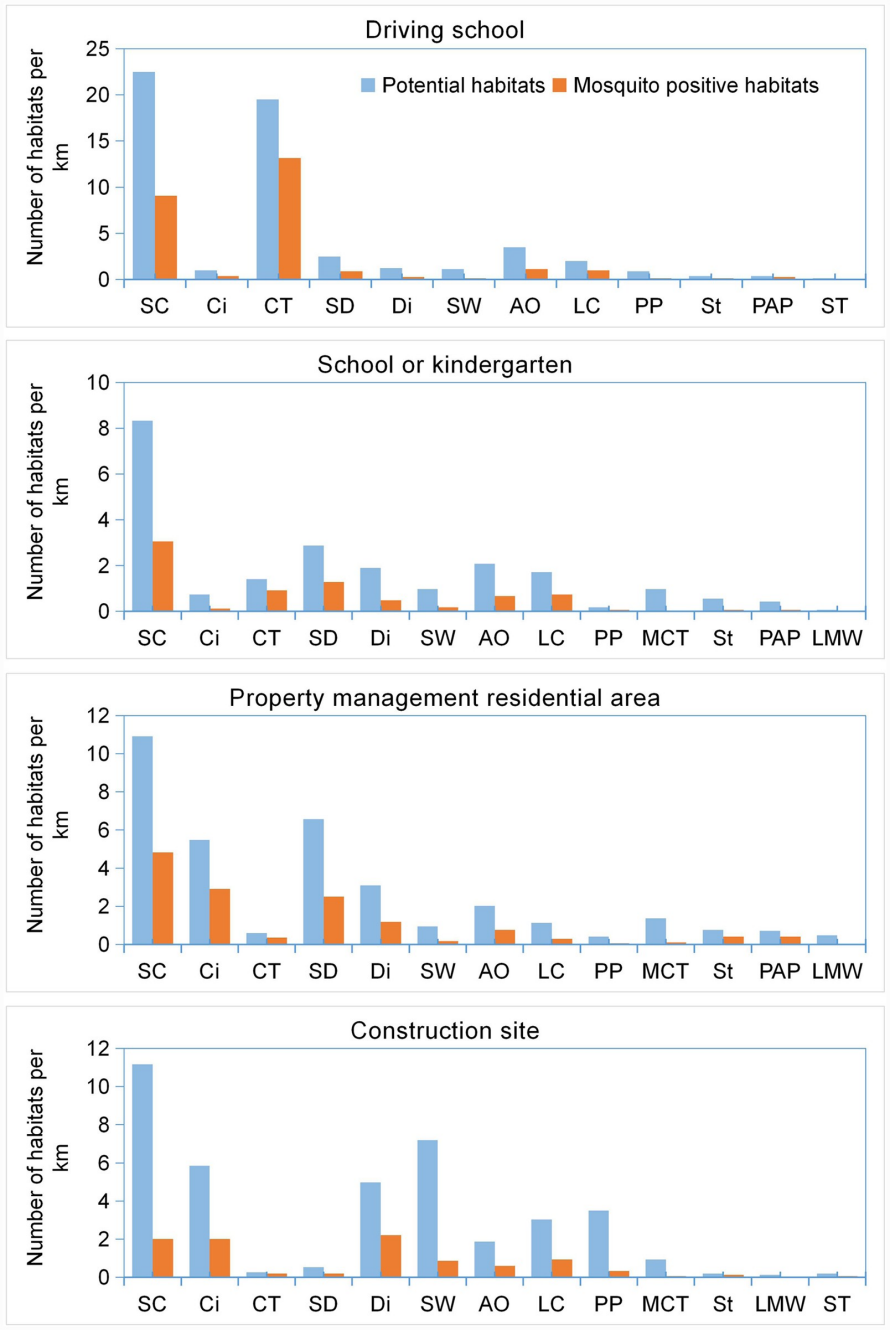
Number of potential habitats and mosquito-positive habitats per km of inspection route by habitat type in built-up areas of Wuhan. That SC, Ci, CT, SD, Di, SW, AO, LC, PP, MCT, St, PAP, LMW and ST were represented by small container, cistern, car tire, storm drain, ditch, shallow water, artificial object, large container, pit or puddle, manhole or cable trench, stump, potted (or aquatic) plant, large and medium water, septic tank, respectively.

Significant differences (P < 0.001) were found in the proportions of potential and mosquito-positive habitat types among the first 4 premise types (Fig 2). There were significantly more cisterns in PMRAs and CSs, more car tires in DSs and SKs, more storm drains in SKs and PMRAs (P < 0.001). In general, the most common potential habitat type was small containers, the average densities of which in DSs, SKs, PMRAs and CSs were 22.5, 8.3, 10.9 and 11.2 /km, respectively. Car tires showed the highest mosquito-positive percentage (67%), followed by potted plants (45%), cisterns (42%), storm drains (39%), stumps (39%), ditches (38%), large containers (36%), small containers (35%), artificial objects (34%), septic tanks (25%), shallow waters (14%), pits and puddles (12%) and manholes or cable trenches (6%). No mosquitoe-positive LMW was found in the first 4 types of premises. In addition, 31 (17.2%) of the 180 storm drains in 36 segments of WRSD and 8 (33%) segments of UC were found positive for immature mosquitoes. DI of the 24 segments of UC was 15% (higher than the grade C criteria, 5%) while the average number of immature mosquitoes was 2.8 (lower than the grade C criteria, 8 immature mosquitoes/positive dip).

The largest and smallest mean route length and elapsed time were 0.465 km and 0.540 hrs in PMRAs and 0.224 km, 0.242 hrs in DSs, respectively (Table 1). Mosquito-positive habitats were not found in 4 DSs, 3 SKs, 3 CSs and 19 segments of WRSD and the rest of the sampled premises were not met with the grade C criteria. The mean CIs, RIs, and TIs were significantly different in the 5 types of premises (P < 0.05). A gradient from (highest) DSs through PMRAs, CSs and SKs to segments of WRSD (lowest) existed for RI and TI, while CI was higher in SKs than in CSs. The mean RIs of DSs, SKs, PMRAs, CSs, and segments of WRSD were 36.9, 10.6, 18.5, 13.3, and 5.3 times higher than the grade C criteria. There were no significant differences between larval indices of central and non-central district in each of the 5 premise types. No differences in larval indices were found among premises in which mosquito management was undertaken by professional pest control companies, by occupants of the premises itself, or by administrative intervened (attending relevant training courses/meetings and rectification was demanded in larval source reduction and these were regarded as administrative intervened, see Table 2) and those with no intervention.

**Table 1:** Length and elapsed time of inspection and the number of premises that met the criteria for vector density control and larval indices of five types of premise in built-up areas of Wuhan.

	Driving school	School or kindergarten	Property management residential area	Construction site	Wide road with storm drain
	(n = 36)	(n = 36)	(n = 36)	(n = 36)	(n = 36)
Mean length of Route (S.E^a^) (km)	0.224(0.015)	0.453(0.029)	0.465(0.024)	0.413(0.023)	0.226(0.014)
Mean elapsed Time (S.E) (h)	0.242(0.011)	0.437(0.024)	0.540(0.022)	0.472(0.026)	0.258(0.010)
Premise met the criteria for vector density control (Mosquito: GB/T 27771–2011), n (%)	4(11.1)	3(8.3)	0(0)	3(8.3)	19(52.8)
Mean CI^b^ (S.E)*	47.9(4.7)A	38.4(4.5)A	41.0(2.4)A	23.5(2.8)B	17.2(4.3)C
Mean RI^c^ (S.E)*	29.5(3.7)A	8.5(1.3)C	14.8(1.1)B	10.6(1.7)C	4.2(1.2)D
Mean TI^d^ (S.E)*	25.2(3.2)A	8.1(1.0)C	12.4(0.9)B	9.0(1.3)C	3.5(1.0)D

*Means with different letters indicate significantly different rank sums (P < 0.05).

^a^. S.E: standard error.

^b^. CI: the percentage of mosquito habitats that are positive for larvae.

^c^. RI: the number of mosquito-positive habitats per km of inspection route.

^d^. TI: the number of mosquito-positive habitats per hour of inspection.

**Table 2:** Mosquito infestation and management supplies, knowledge, practices, the PHCS’ guidance and supervision surrounding NSC mosquito management of built-up areas of Wuhan.

	Driving school	School or kindergarten	Property management residential area	Construction site	Total
	(n = 36)	(n = 36)	(n = 36)	(n = 34)	(n = 142)
**Mosquito infestation in the premise, n (%)**					
the mosquitoes were worse at that time than the last 2 years					
Yes	1(3)	0(0)	2(6)	1(3)	4(3)
No, they were better	2(6)	5(14)	7(19)	3(9)	17(12)
It seemed to be no difference	28(78)	31(86)	24(67)	26(76)	109(77)
Did not know	5(14)	0(0)	3(8)	4(12)	12(8)
Equipped with mosquito management supplies, n (%)*					
Flashlight	0(0)	16(44)	21(58)	7(21)	44(31)
Mosquito dipper	0(0)	11(31)	4(11)	2(6)	17(12)
Grate lifting hook	0(0)	9(25)	14(39)	4(12)	27(19)
Sprayer	13(36)	27(75)	36(100)	21(62)	97(68)
Larvicide recommended by the PHCCO	0(0)	6(17)	3(8)	2(6)	11(8)
Adulticide					
Adulticide recommended by the PHCCO	13(36)	23(64)	11(31)	8(24)	55(39)
DDVP	2(6)	0(0)	19(53)	16(47)	37(26)
**Knowledge, n (%)**					
Had heard about Wuhan urban pest management regulations	2(6)	1(3)	7(19)	3(9)	13(9)
Mosquito management responsibility of the premise					
Knew the PHCCO’s arrangement of the responsibility	17(47)	35(97)	36(100)	25(74)	113(80)
Did not know	19(53)	1(3)	0(0)	9(26)	29(20)
Knew that larval source reduction is the most suitable mosquito management strategy	4(11)	7(19)	7(19)	3(9)	21(15)
**Practices, n (%)**					
Who did mosquito management					
Contracted with a professional pest control company	0(0)	11(31)	5(14)	2(6)	18(13)
Personnel of the premise	15(42)	24(67)	31(86)	28(82)	98(69)
Nobody	21(58)	1(3)	0(0)	4(12)	26(18)
Indoor or outdoor spraying*	13(36)	27(75)	36(100)	21(62)	97(68)
Larval source reduction*	3(8)	9(25)	5(14)	3(9)	20(14)
**Had received the PHCS’ guidance and supervision on mosquito management, n (%)**					
Premise personnel had attended relevant training course/meeting	0(0)	22(61)	19(53)	9(26)	50(35)
Premise personnel had got electronic/paper edition of					
Wuhan urban pest management regulations	0(0)	0(0)	4(11)	0(0)	4(3)
CNSC vector management specifications	0(0)	8(22)	6(17)	5(15)	19(13)
Visited by health inspectors and positive mosquito habitats were found in the premise and was demanded rectification in larval source reduction					
In oral form	2(6)	5(14)	8(22)	9(26)	24(17)
In written form^a^	0(0)	2(6)	3(8)	2(6)	7(5)

*The contracted professional pest control companies equipped with a certain supply and carried out a certain practice was calculated as the premise equipped with that supply and carried out that practice, respectively.

^a^. Received an official notification or bulletin for rectification.

### Administrative intervention of responsible departments

Seventy seven percent of premise occupants responded that there is no difference between the mosquito infestation in their premises at the time of the survey and before 2 years. All the PMRAs and more than half of the SKs and CSs were equipped with sprayers (including backpack, hand and aerosol sprayers). About a quarter of the respondents (mainly of PMRAs and CSs) reported that they used DDVP (dichlorvos), even if it was banned by the PHCCO for mosquito management. Only 8% of premises were equipped with larvicides recommended by the PHCCO. Nine percent respondents heard about WUPMR. Eighty percent of the respondents was aware of the responsibility and arrangements of PHC on mosquito management in their premises, however 53% of the respondents of DSs was not aware of it. Knowledge of the respondents on the larval source reduction strategy was very poor (15%). Mosquito management in most of the premises (69%) was done by the occupants themselves and only a few premises (13%) reported that they contracted with a professional pest control company for mosquito management. The main action against mosquitoes was indoor or outdoor spraying (in 68% premises), while larval source reduction was not carried out by many (only in 14% of premises). More than half of SKs, PMRAs have attended relevant training courses/meetings held by the PHCS since 2013, whereas none of the DSs. Only 3% and 13% of premises had received electronic/paper edition of WUPMR and CNSCVMS from the PHCS respectively. Seventeen percent of the premises have received oral demands from the health inspectors to rectify mosquito-positive habitats and only 5% received an official notification or bulletin for rectification (Table 2).

Seventy four percent of the responsible departments endorsed their mosquito management responsibility specified by WUPMR and PHC documents. Water Authorities had shown 100% endorsement followed by Construction Management Stations (92%) and Education Bureaus (81%). The majority of Sub-district Offices which were responsible for mosquito management in DSs had not endorsed the responsibility and claimed that DSs were small private enterprises that could not be managed by them. According to some district Education Bureaus, private and/or municipal SKs sampled in this study were out of their jurisdictions. A few Construction Management Stations deemed that they were not responsible for vector management of municipal and provincial CSs in their districts. About half of the responsible departments had held training courses/meetings related to CNSC vector management from 2013 to 2015 although they could not confirm the participation of all premises under their jurisdiction. Distribution of CNSCVMS and WUPMR to premises by the responsible departments was very poor (25% and 5% respectively). About a quarter of responsible departments reported that they had received the official notifications or bulletins from superior agencies of PHCS for mosquito management of the premises under their jurisdiction. Only 40% of the departments had carried out on-site supervisions accompany with PHCS experts and/or officers (Table 3). The majority of responsible departments reported that they had not been trained for identifying mosquito breeding habitats and they had not supervised their premises’ mosquito management on site independently.

**Table 3:** Guidance and supervision of district responsible departments surrounding NSC mosquito management of built-up areas of Wuhan.

	Sub-district Office	Education Bureau	Housing Management Bureau	Construction Management Station	Water Authority	Total
	(n = 36)	(n = 36)	(n = 36)	(n = 36)	(n = 60)	(n = 204)
Endorsed responsibility for supervision, n (%)	4(11)	29(81)	25(69)	33(92)	60(100)	151(74)
	(n = 31)	(n = 11)	(n = 12)	(n = 10)	(n = 11)	(n = 75)
Hold relevant training course/meeting, n (%)	0(0)	11(100)	10(83)	10(100)	10(91)	41(55)
Provided electronic/paper edition of, n (%)						
Wuhan urban pest management regulations	0(0)	1(9)	3(25)	0(0)	0(0)	4(5)
CNSC vector management specifications	0(0)	6(55)	6(50)	5(50)	2(18)	19(25)
Had received an official notification or bulletin for their supervised premises’ mosquito management rectification, n (%)	2(6)	3(27)	4(33)	5(50)	4(36)	18(24)
Had supervised premises’ mosquito management on site, n (%)						
Accompany with PHCS experts and/or officers	2(6)	8(73)	9(75)	6(60)	5(45)	30(40)
Independently	0(0)	2(18)	0(0)	1(10)	0(0)	3(4)

## Discussion

All the larval indices (total RI) indicate that the mosquito habitat density in the Wuhan city was considerably high and did not meet the grade C criteria for vector density control of NSC. As implied by the questionnaire survey of premises, mosquito management in almost none of the sampled premises was done according to the expectations of the CNSC program. Although most of the premises had awareness about the PHC and its responsibilities they had not received much guidance in mosquito management. The officials of the PHCS basically did not provide the trainings, electronic version, official notifications for rectification of mosquito habitats. Majority of the premises carried out mosquito management by themselves supposing indoor/outdoor spraying should be the method of choice. Although the CNSC had emphasized on larval source reduction only a few of the premises had adopted it and there were premises which had not been doing any type of mosquito management activities.

Although a high proportion of responsible departments had seemingly endorsed their responsibility in mosquito management of those premises, their active engagement was not satisfactory. Most of the departments admitted that many activities such as distribution of CNSCVMS and WUPMR, distribution of bulletins, carrying out on-site supervision of premises with experts had been neglected. Despite 55% of the departments had held trainings/meetings, most of the relevant premise occupants claimed that they did not have appropriate training to carry out the work properly. It was reported that some of them sprayed insecticides to part of their WRSDs each year without knowing which segments were found mosquito-positive [29]. They did not even know if the sprayed WRSDs had stagnant waters nor they had any idea about the sprayed insecticides. The operations were far from integrated vector management [30,31]. In July 2014, a segment of UC in Qingshan district was found to have a huge number of immature mosquitoes by the third-party unannounced visit. The district Water Authority was not capable to deal with it and the service of a pest control company was requested by the district PHCCO. Results show that mosquito management of urban Wuhan was far from the grade C criteria [32]. There is evidence that similar defects of larval source reduction or mosquito management [33,34] existed in urban areas of other NSCs of Central and South China [22]. Those defects do not fit to the recent socioeconomic status [35] of Chinese cities, and it is the violation of the original intention of the National PHCCO in organizing CNSC activities. However, Wuhan passed the NSC assessment review organized by the National PHCCO with much fewer efforts in 2017. The main reasons for poor mosquito management cities to go through National and Provincial PHCCO’s NSC assessments could be summarized as follows:

1. Technical assessments by the national and provincial experts are carried out by using pre-selected samples of premises instead of taking random samples as specified by the NSC standards.
2. Experts have inspected much larger samples, than normal people can do, in a very short period of time. They have not inspected sanitary corners and far ends of backyards of the premises in which the mosquito habitats are commonly found.
3. The National PHCCO assessments every 3 years after a city is awarded NSC title (during period of Consolidating NSC) are less rigorous than CNSC assessments. Both assessments have insufficient consideration of the sustainable mosquito management mechanism of the NSC.

Findings of the study show that the administrative mechanisms of the PHCS of the city was below the expectations of the CNSC program. Administrative pressure is essential for premises to carry out good mosquito management practices, especially larval source reduction [36,37]. Although premises with low social status (e.g. small private enterprises like DSs) are generally ignorant of rectification requirements. According to our experience, demanding rectification in the form of official notification or bulletin improves mosquito management of premises with certain social status (e.g. government agencies, well-known schools and enterprises) [38]. Achieving the criteria for mosquito management requires a lot of administrative intervention and technical work. However, law enforcement in mosquito management is often not feasible in mainland China. Therefore, we suggest that, at first place, the PHC Committee of Wuhan administration related to mosquito management should be strengthened by clarification and endorsement of administrative responsibilities [39] of each component of the PHCS. We believe that many NSCs have the ability and capacity to do better mosquito management [40]. NSC standards should be strictly adhered in mosquito management by responsible administrative departments, as well as the premises. Strict adherence to the NSC standards by assessing experts is required in order to do credible urban mosquito management assessments. But before that, we need more scientific and reasonable NSC standards. More powerful weapons such as mosquito-related legislation and law enforcement [8], which China needs to learn from western developed countries, should be introduced in the assessments of the sanitary city evaluation. The assessments need more sustainable mosquito management mechanism of NSC verifications. Increasing governmental funds for training professionals and mosquito management practice in the premises with low social status, like Haikou and Ningbo cities are recommended [41].

## Conclusions

Mosquito management in the premises of built-up areas of Wuhan was below the levels expected by CNSC program. The administrative interventions of the PHCS of the city had not evidently improved mosquito management in those premises. The RI was much higher than the grade C criteria, shortly after the success of CNSC. Administration is the weak link of urban integrated mosquito management in the city [42]. To combat mosquito-borne diseases [43,44] and to improve the quality of life of residents, we recommend that PHCS honestly reveals defects in urban mosquito management and seriously takes those exposed defects. The PHCS should strengthen PHC activities by strict adherence to NSC standards. A mechanism should be established to monitor the activities carried out by responsible departments as well as premises [45]. Further research on sustained promotion of urban mosquito management of NSCs, which focus on effective administration, as well as on improvement of related sectors of NSC standards are needed.

## Supporting information

S1 Fig Map of Wuhan showing the scope of CNSC program. (JPG)

S2 FigLocations of the study sites for larval surveys in built-up areas of Wuhan.Symbols: ○ = driving school; ○ = school or kindergarten; ○ = property management residential area; ● = construction site; ● = wide road with storm drain; ● = urban creek.(TIF)

## Abbreviations

CI: container index
CNSC: Creating National Sanitary City
CNSCVMS: CNSC vector management specifications
CS: construction site
DI: dip index
DS: driving school
EFE: eliminating the Four Evils
LMW: large and medium water
NSC: National Sanitary City
PHC: Patriotic Health Campaign
PHCCO: PHC Committee Office
PHCS: PHC system
PMRA: property management residential area
RI: route index
SK: school or kindergarten
TI: time index
UC: urban creek
WRSD: wide road with storm drain
WUPMR: Wuhan urban pest management regulations
