# Supplementary figures and images for: Urban mosquito management administration: Mosquito (Diptera: Culicidae) habitat surveillance and questionnaire survey in Wuhan, Central China
Source: PLoS One. 2020 May 5;15(5):e0232286. doi: 10.1371/journal.pone.0232286 (PMC7199959; doi:10.1371/journal.pone.0232286)

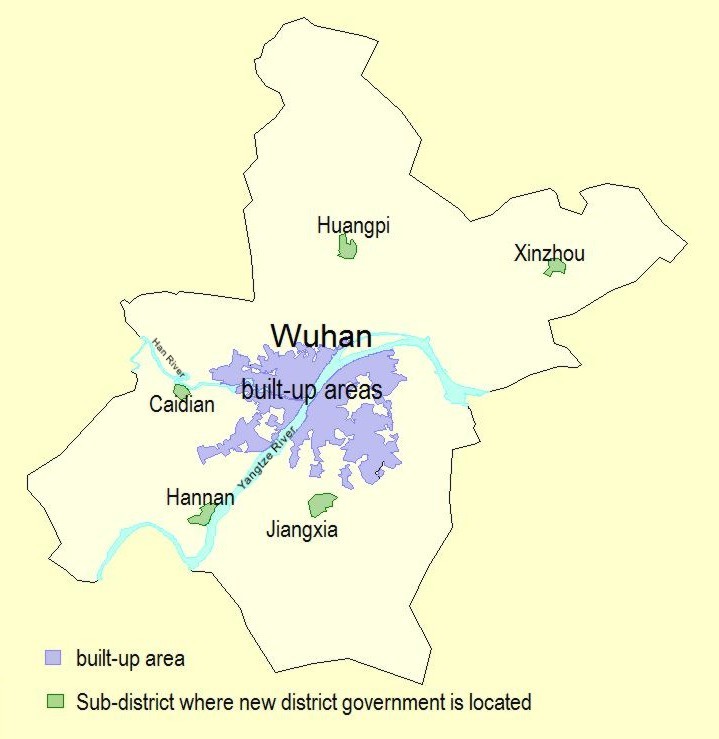

Supplement: S1 Fig — (JPG) [file pone.0232286.s001.jpg]

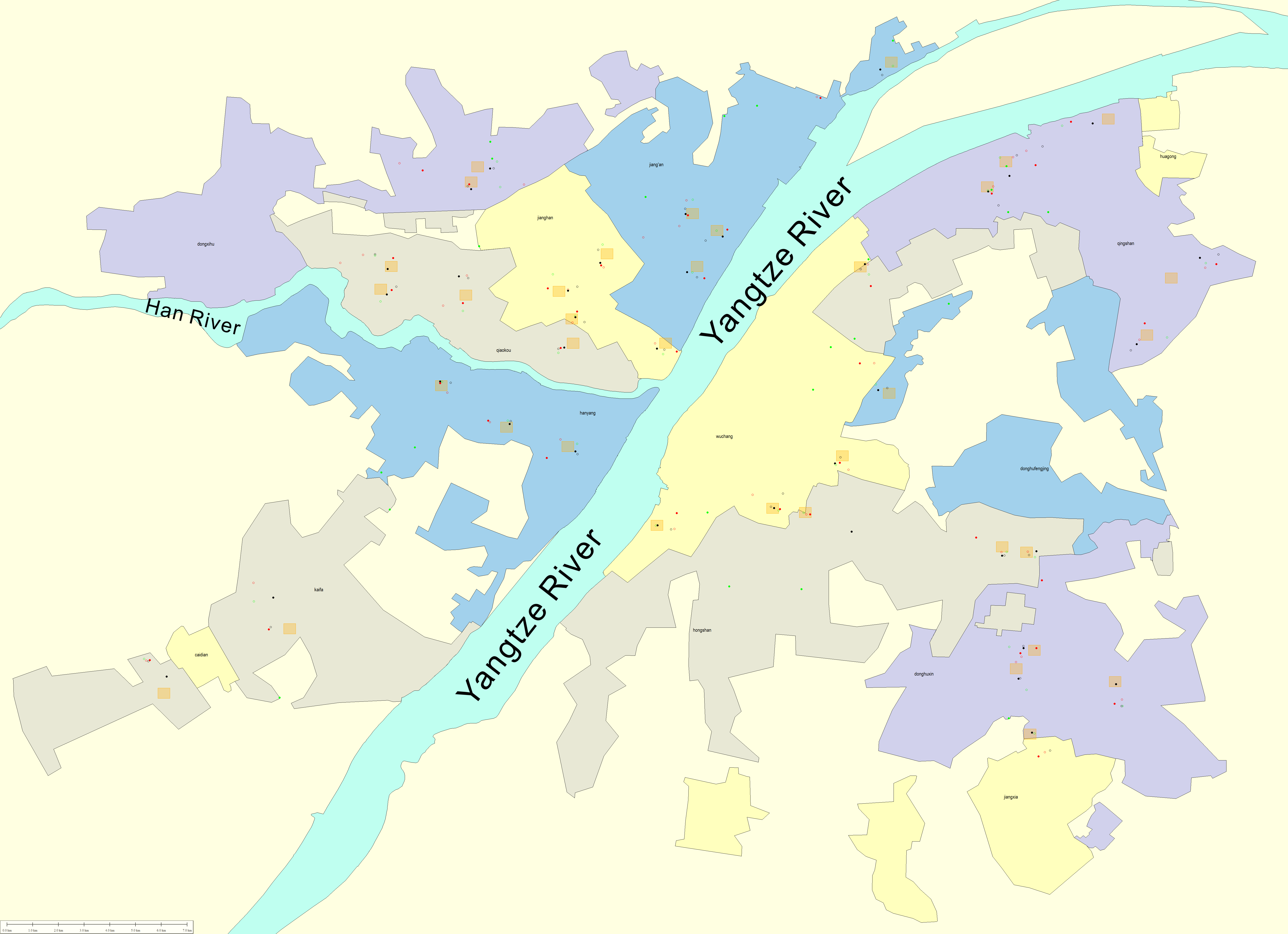

Supplement: S2 Fig — Symbols: ○ = driving school; ○ = school or kindergarten; ○ = property management residential area; ● = construction site; ● = wide road with storm drain; ● = urban creek. (TIF) [file pone.0232286.s002.tif]
